# Effect of sulfur on pollen germination of Clemenules mandarin and Nova tangelo

**DOI:** 10.7717/peerj.14775

**Published:** 2023-02-07

**Authors:** Roberto Beltrán, Nuria Cebrián, Carlos Zornoza, Francisco García Breijo, José Reig Armiñana, Alfonso Garmendia, Hugo Merle

**Affiliations:** 1 Departamento de Ecosistemas Agroforestales, Universitat Politècnica de València, Valencia, Spain; 2 Instituto Cavanilles de Biodiversidad y Biología Evolutiva, Universidad de Valencia, Valencia, Spain; 3 Instituto Agroforestal Mediterráneo, Universitat Politècnica de València, Valencia, Spain

**Keywords:** Copper sulfate, Ammonium sulfate, Pollination, Pollen tube, Mandarin, Inorganic sulfur

## Abstract

This study aims to elucidate whether sulfur can inhibit citrus pollination by affecting pollen grains. For this, four sulfur-based products (inorganic sulfur, water dispersible granular sulfur, ammonium sulfate, copper sulfate) were tested to evaluate their effect on pollen germination and pollen tube growth of two citrus varieties: Clemenules mandarin (*Citrus clementina*) and Nova tangelo (*Citrus clementina* x [*Citrus paradisi* x *Citrus reticulata*]). Pollen grains were extracted from the flowers of these two varieties and subsequently placed in Petri dishes with modified BK (boron and potassium) germination medium with six concentrations of the sulfur-based products (0.2, 2, 20, 200, 2,000, 20,000 mg l^−1^). All the dishes were incubated and the pollen germination rate was calculated. All the sulfur products showed progressive pollen germination inhibition with a rising sulfur concentration. CTC50 (50% cytotoxicity inhibition) was around 20 mg l^−1^, with significant differences among treatments. Total pollen germination inhibition took place at 20,000 mg l^−1^. These results demonstrate that sulfur application can affect citrus pollination.

## Introduction

Citrus varieties show diverse botanical and physiological characteristics. Some varieties present self-pollination or cross-pollination that results in ovule fertilization and seed formation (Mesejo et al., 2013). Conversely as several varieties are self-incompatible, they do not have self-fertilization. In these cases, fruit are parthenocarpic and the seeds that appear in them are due to crosses with pollen from other compatible varieties (Gambetta et al., 2013). Several studies indicated that ovule fertilization with pollen and subsequent seed formation was directly related to variety’s yield. Chao (2005) reported how citrus varieties with cross-pollination and seed formation had larger fruit than seedless varieties. Self-incompatibility and parthenocarpy were related with lower yields because seeds were absent (Yamamoto, Kubo & Tominaga, 2006).

In some cases, obtaining seedless varieties has been a sought-after character to increase the commercial value of fruit. In any case, it should be taken into account that limitation of fertilization or alteration of natural pollination can lead to the opposite situation: loss of both fruit size and yield. Likewise, studies on pollen tube kinetics are essential because flower fertilization and appearance of seeds depend on it (Mesejo et al., 2007). The effect that several natural or synthetic chemical compounds can have on the entire pollination and fertilization process is very important for its implications on production. Sometimes the effect of some products on the ovule or pollen-bearing vehicles has been tested rather than on pollen itself. Most of the studies carried out to date have focused on the effect that pesticides might have on pollination (Richards, 2001). Some studies have been done on other substances. Garmendia et al. (2019) observed that some insect repellents had no effect on the cross-fertilization of the ‘Nadorcott’ mandarin and did not, therefore, avoid ovule fertilization and seed formation.

It is well-known that gamma ray irradiation, used to obtain seedless citrus varieties, alters the germination and characteristics of pollen (Bermejo, Pardo & Cano, 2011). This contrasts with the lack of information about the effect that other agricultural practices may have on pollen tube growth and pollen germination. Indeed, very few studies are available on the effect that various substances might have on the pollen fertilization and seed formation of some citrus varieties. An inhibitory effect on pollen tube growth and pollen germination in ‘Nadorcott’ mandarins after applying copper sulfate was observed (Mesejo et al., 2006). This reduction in pollen germination produced fewer seeds. Instead, a similar study conducted in Uruguay also with ‘Nadorcott’ mandarin pointed out a minor reduction in pollen tube growth after applying copper sulfate and gibberellic acid (Gambetta et al., 2013). In this experiment, pollen tubes almost reached their normal length and less seed reduction took place. Both experiments were carried out *in vivo* under field conditions, where other factors may have had an influence, which could explain the apparent contradiction in the results. In addition, these works did not clearly indicate if the potential effect of this substance was due to copper or to sulfur. In a recent study conducted on ‘Nadorcott’ mandarin flowers, sulfur was found to inhibit pollen tube growth through stigma tissue (Garmendia et al., 2022a). These observations made us wonder about the mechanism by which sulfur reduces the number of seeds. It would also be very interesting to know if sulfur affects the tissues and exudate of the stigma or if it alters the germination of pollen grains. A study *in vitro* of how the pollen of citrus varieties compatible with ‘Nadorcott’ mandarin behaves would help us understand this mechanism. On the other hand, for citrus growers it is important to know the effect of certain substances that could potentially prevent or hinder pollen germination, especially with substances frequently used in agricultural practices. The use of sulfur in soil fertilization has increased in recent years (Lucheta & Lambais, 2012). According to some of the above-cited studies, the effect of some sulfur-based products on pollination has been tested, but information is still scarce, and some questions arise that should be answered. So, the effects that have been observed in the germination of these citrus varieties does occur in the female part (stigma) or in the male part (pollen)? What is the chemical form of sulfur in which these effects are best observed? This last question is interesting because in some of the references found sulfur was used in the form of inorganic sulfate, but in others it was in another inorganic form. The present work is a continuation of the study performed by Garmendia et al. (2022a), and the purpose is to try to answer the questions posed before. Therefore, we proposed an *in vitro* study to find out if sulfur is capable to inhibit pollen germination, which would help explain the results obtained in that study. For this reason, this study will be carried out with pollen of mandarin varieties compatible with ‘Nadorcott’, since this is the type of pollen that we found in the stigma of ‘Nadorcott’ flowers and which is capable of fertilizing the ovary and producing seeds. Different products in which sulfur can be found in inorganic form and in inorganic sulfate form will be tested to find out which of them is more effective.

## Materials and Methods

### Plant material

Fresh pollen from two citrus varieties compatible with ‘Nadorcott’ mandarin, Clemenules mandarin (*Citrus clementina* hort.) and Nova tangelo (*Citrus clementina* hort. x [*Citrus paradisi* Macfad. x *Citrus reticulata* Blanco]), was used for the experiments. Flowers were collected from March to April 2017. Sampling was performed early in the morning with flowers at anthesis. The sampled flowers were stored in a refrigerator (4 °C) being processed before 24 h to avoid loss of viability.

Flowers came from adult trees growing in a commercial orchard located in Montserrat (Valencia Province, Spain; Latitude: N 39.359629, Longitude: E-0.547494, Altitude: 153 m). The site’s climate is Mediterranean with an average temperature of 16.8 °C and average rainfall of 432 mm.

### Treatments

The sampled flowers were left inside a humid chamber (4 °C, 2 h) for prehydration before extracting pollen (Mesejo et al., 2006). A modified (boron + potassium) BK medium was prepared to induce pollen germination (Beltrán et al., 2020). It contained 100 g l^−1^ sucrose, 0.1 g l^−1^ H_3_BO_3_, 0.3 g l^−1^ Ca(NO_3_)_2_, 0.1 g l^−1^ KNO_3_ and 10 g l^−1^ agarose. All ingredients were mixed and autoclaved at 120 °C for 1 h. Subsequently, the medium was dropped into 90-mm Petri dishes (5 ml of medium per dish) using a flow cabinet to avoid contamination. This BK medium was the positive control, in which pollen should germinate normally. In addition, four treatments consisting of sulfur-based products in which we can find sulfur in two chemical forms were tested: inorganic sulfur (IS); water dispersible granular sulfur (GS); ammonium sulfate (NS); copper sulfate (CS). BK medium was the base of these treatments, to which each of these substances was added. These treatments were prepared in the same way as the previous one, using an autoclave and a flow cabinet to ensure sterility. Six progressively descending dilutions were performed for these four treatments. In the case of IS, dilutions started from 20,000 mg l^-1^ and went to 0.2 mg l^−1^. Also, a surfactant (Tween©) was added to improve its dissolution. For the other sulfur-based products, the equivalent weight of sulfur in the initial dilution needed to obtain 20,000 mg l^−1^ was calculated (25,000 mg of GS, 125,000 mg of NS, 90,000 mg of CS). Then successive dilutions were carried out until the last dilution of 0.2 mg l^−1^. All plates were stored in a refrigerator prior to use.

### Germination and measures

**Figure 1:**
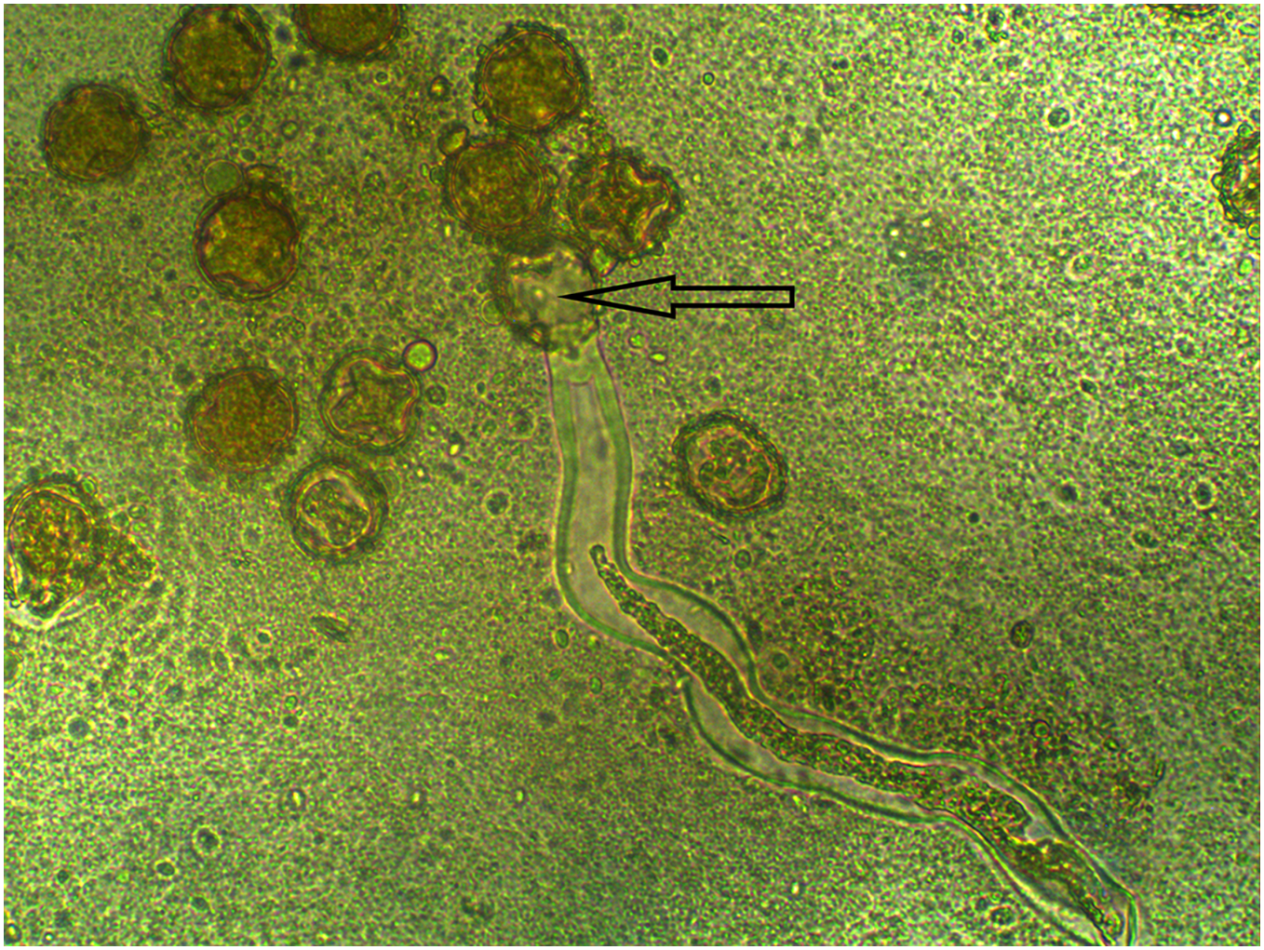
Pollen grains of Nova citrus variety. The arrow indicates a germinated pollen grain, whose pollen tube exceeds several times the diameter of the pollen grain.

Pollen grains were extracted from the anthers of both citrus varieties using a stereo microscope (Optika, Bergamo, Italy, 4× magnification). They were placed in the Petri dishes containing the corresponding treatment. These steps were performed in a flow cabinet to avoid contamination by spores, which could distort the results and make their evaluation more difficult. Dishes were left in an incubator for 72 h in the dark at 25 °C for germination purposes (Beltrán et al., 2019). Five dishes per treatment and concentration were sown per citrus variety. Evaluation was carried out 4 days after every sowing. An optical microscope (Leica, Wetzlar, Germany, 40× magnification) was used to observe the pollen grains and its pollen tube. In each dish, 100 pollen grains were randomly searched to check if they were germinated or not. Pollen was considered germinated when pollen tube length exceeded the diameter of its pollen grain (Fig. 1). The pollen germination percentage (PPG) of each treatment and dilution was calculated as the mean percentage of pollen germination in all the repetitions. The inhibition effect of treatments on pollen germination was calculated by comparing the germination treatment with the positive control germination from the same repetition. The response model of the different treatments carried out in Clemenules and Nova on the pollen germination rate was calculated by plotting a curve for each tested product adjusted to the pollen germination rates per dose. Finally, the estimated CTC50 parameter (effective dose in mg l^−1^ for 50% pollen germination inhibition) was calculated. The whole experiment was repeated three times, which totaled 720 sown dishes.

### Statistical analyses

The mean, standard error and skew of PPG values were calculated. ANOVA and Tukey *post hoc* tests were used to compare the values between treatments and concentrations. In some cases, due to the lack of normality, nonparametric methods were used to compare among treatments and concentrations by a Kruskal–Wallis test with Holm correction in the *post hoc* test. Levene’s test and eta-squared statistics were calculated if significant differences were found. The analyses, tables and figures were created in R language (R Core Team, 2017) using RStudio gui (RStudio Team, 2016). Other employed packages were “agricolae” (Mendiburu, 2019) for the *post hoc* tests and “ggplot2” (Wickham, 2020) for graphics. For the estimation of the CTC50 and the elaboration of its related figures, “drc” package was used (Ritz et al., 2015).

## Results

**Figure 2:**
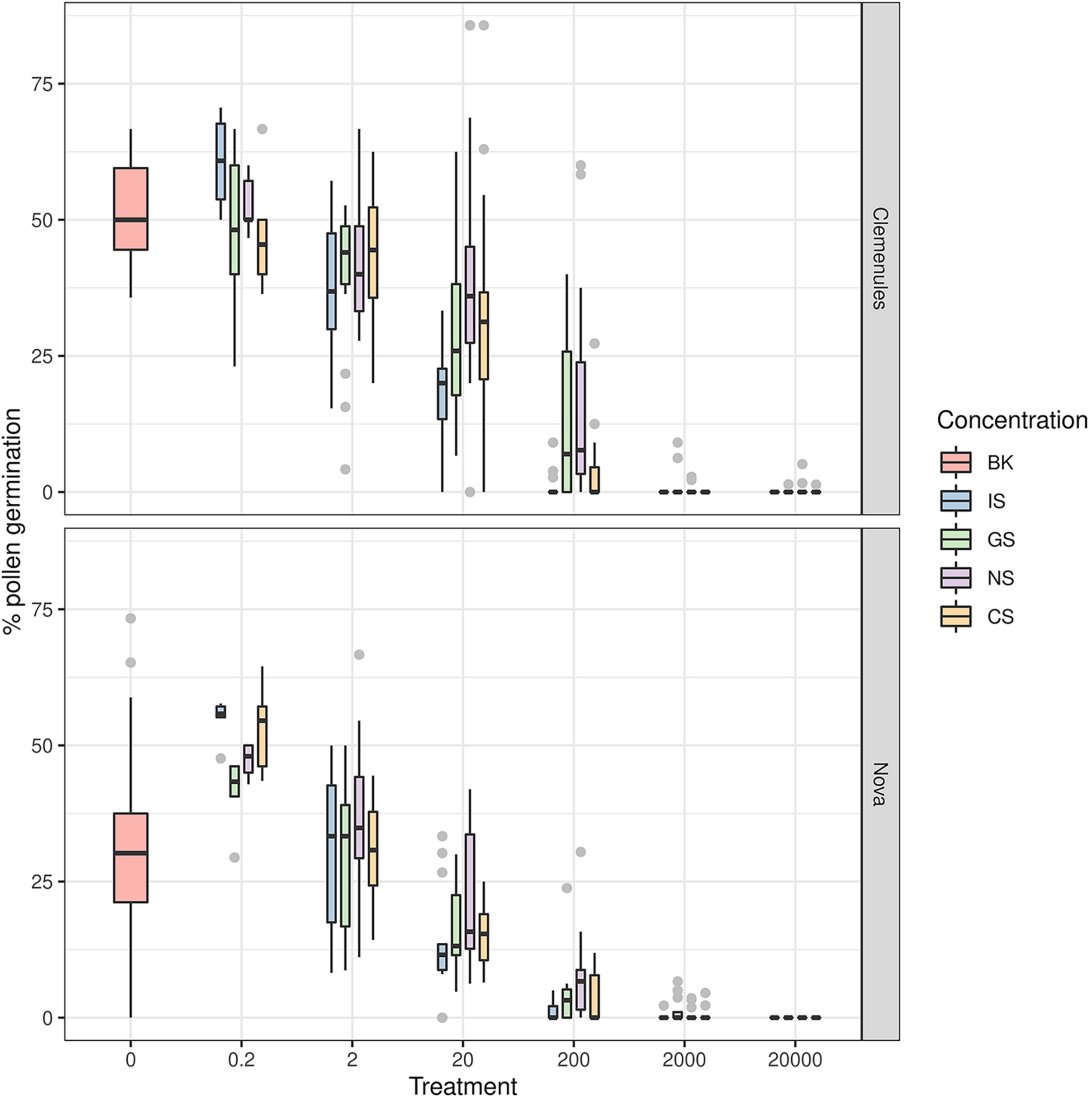
Effect of sulfur treatments on the pollen germination rate for the six tested concentrations with Nova and Clemenules citrus varieties. Boxes show the 25th and the 75th percentiles. Lines in the boxes show the median values. The whiskers indicate the maximum and the minimum values. The gray circles are the outlayers. Treatments: BK, positive control; CS, copper sulfate; NS, ammonium sulfate; IS, inorganic sulfur; GS, water dispersible granular sulfur.

Both varieties showed a lower PPG as the sulfur concentration increased (Fig. 2). The boxplots for Clemenules mandarin and Nova tangelo highlight that no significant differences were observed by applying the different chemical form of all S-compounds, at parity of dose. These results indicated that dose is more important than that of the chemical form. For Clemenules, the treatments with the lowest sulfur concentration (0.2 mg l^−1^) showed the highest PPG (ranging from 47.57% for GS to 60.56% for IS). For this concentration there were no significant differences with the positive control (BK treatment, 52.09 ± 2.57%). The lowest PPGs (close to zero) were obtained for the treatments with the highest sulfur concentrations (2,000 and 20,000 mg l^−1^), which showed a large significant difference with the positive control (Table 1).

**Table 1:** Statistics of pollen germination percentages on Clemenules citrus variety.

Treatment	Concentration	Mean	SD	se
BK	0	52.10	9.94	2.57
CS	0.2	47.70	11.81	5.28
CS	2	43.40	12.62	3.26
CS	20	33.27	21.29	5.50
CS	200	4.33	7.41	1.91
CS	2,000	0.00	0.00	0.00
CS	20,000	0.14	0.43	0.14
NS	0.2	52.76	5.57	2.49
NS	2	42.57	12.34	3.19
NS	20	38.78	21.07	5.63
NS	200	17.27	20.24	5.23
NS	2,000	0.52	1.08	0.28
NS	20,000	0.67	1.64	0.52
IS	0.2	60.56	9.67	4.83
IS	2	37.82	12.53	3.24
IS	20	18.38	7.69	1.99
IS	200	1.04	2.51	0.65
IS	2,000	0.00	0.00	0.00
IS	20,000	0.00	0.00	0.00
GS	0.2	47.57	17.15	7.67
GS	2	39.41	14.28	3.69
GS	20	28.56	15.05	3.89
GS	200	2.58	15.89	4.10
GS	2,000	1.02	2.75	0.71
GS	20,000	0.15	0.44	0.14

**Note:**

Treatments: CS, copper sulfate; NS, ammonium sulfate; IS, inorganic sulfur; GS, water dispersible granular sulfur.

Similarly for Nova, the treatments with the lowest sulfur concentration (0.2 mg l^−1^) obtained the highest PPG, which ranged from 41.14% for GS to 54.69% for IS. The positive control obtained a lower PPG (30.65 ± 4.87%, Table 2). The treatments with 20,000 mg l^−1^ of sulfur did not show pollen germination (Table 2). The highest PPGs for Clemenules were registered for IS and NS at 0.2 mg l^−1^ (lowest sulfur concentration), respectively (60.56% and 52.76%) and, in this case, the germination rate of the positive control was similar to these (52.09%). The germination rates lowered for the sulfur treatments as the sulfur concentration rose. IS was the most effective product because total germination inhibition was achieved at the 200 mg l^−1^ concentration. The highest PPGs for Nova were also achieved for treatments CS and IS at the lowest concentration (0.2 mg l^−1^) with 53.17% and 54.69%, respectively. The positive control (BK germination medium only) reached 30.65% of germinated pollen grains, with a higher standard deviation. In all the sulfur treatments, the highest percentage of germinated pollen grains was observed for the lowest concentration (0.2 mg l^−1^). The lowest PPG was achieved for the highest sulfur concentration (20,000 mg l^−1^) because pollen germination inhibition was total in all these cases.

**Table 2:** Statistics of pollen germination percentages on Nova citrus variety.

Treatment	Concentration	Mean	SD	Se
BK	0	30.65	21.80	4.87
CS	0.2	53.17	8.51	3.81
CS	2	30.15	9.89	2.55
CS	20	15.06	5.96	1.54
CS	200	3.63	4.44	1.15
CS	2,000	0.45	1.27	0.33
CS	20,000	0.00	0.00	0.00
NS	0.2	47.17	3.16	1.41
NS	2	35.81	14.91	3.99
NS	20	21.30	12.23	3.16
NS	200	7.44	8.01	2.07
NS	2,000	0.59	1.26	0.32
NS	20,000	0.00	0.00	0.00
IS	0.2	54.69	4.08	1.82
IS	2	30.15	14.33	3.70
IS	20	12.82	10.20	2.63
IS	200	1.13	1.80	0.47
IS	2,000	0.15	0.57	0.15
IS	20,000	0.00	0.00	0.00
GS	0.2	41.14	6.94	3.11
GS	2	29.88	13.96	3.60
GS	20	15.77	7.94	2.05
GS	200	3.84	6.08	1.57
GS	2,000	1.61	2.16	0.56
GS	20,000	0.00	0.00	0.00

**Note:**

Treatments: CS, copper sulfate; NS, ammonium sulfate; IS, inorganic sulfur; GS, water dispersible granular sulfur.

**Figure 3:**
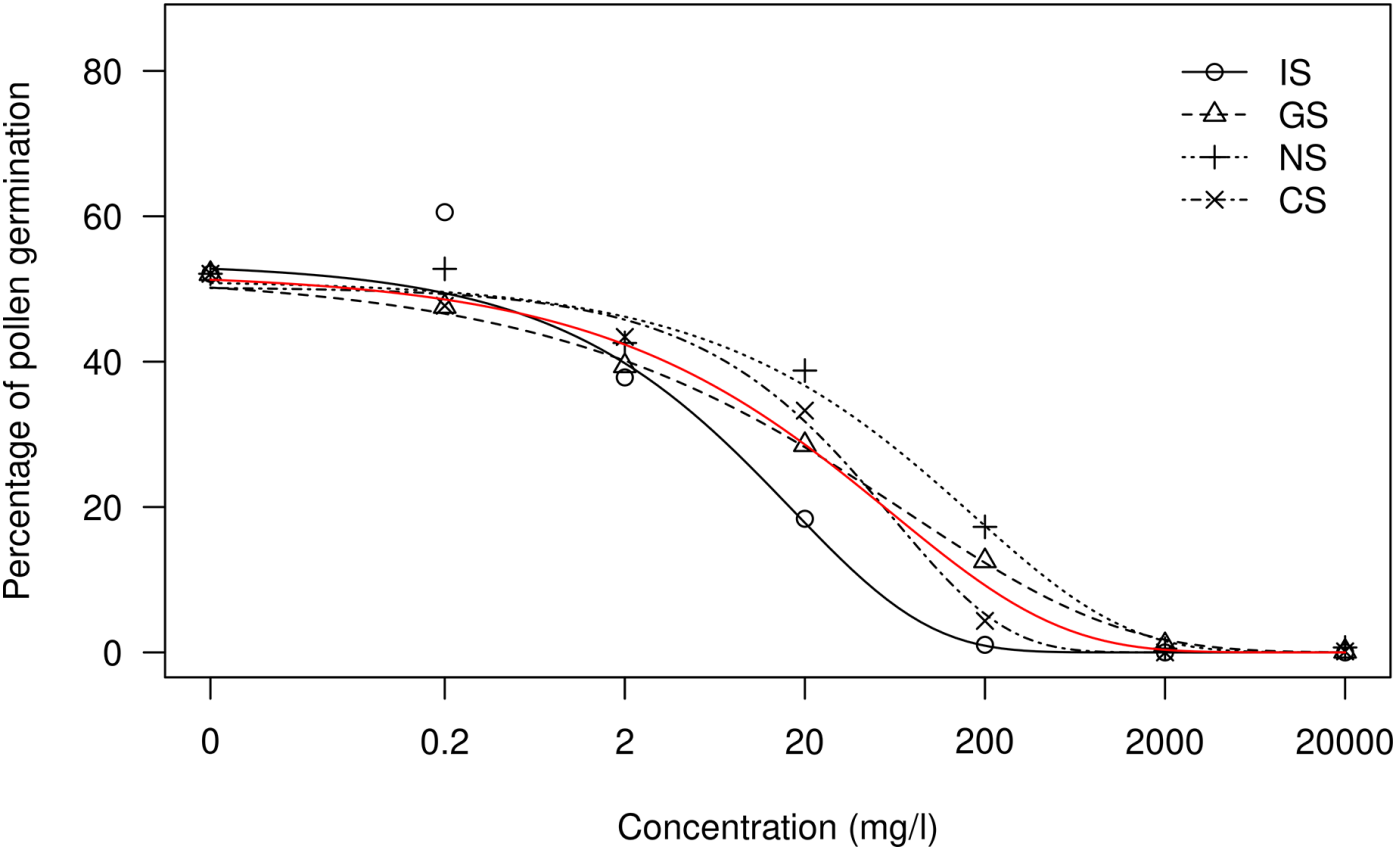
Comparison of the dose-response treatments for the Clemenules citrus variety. Each point represents the mean value for every treatment and concentration. The red line represents the model for all the treatments together. Treatments: BK, positive control; CS, copper sulfate; NS, ammonium sulfate; IS, inorganic sulfur; GS, water dispersible granular sulfur.

**Figure 4:**
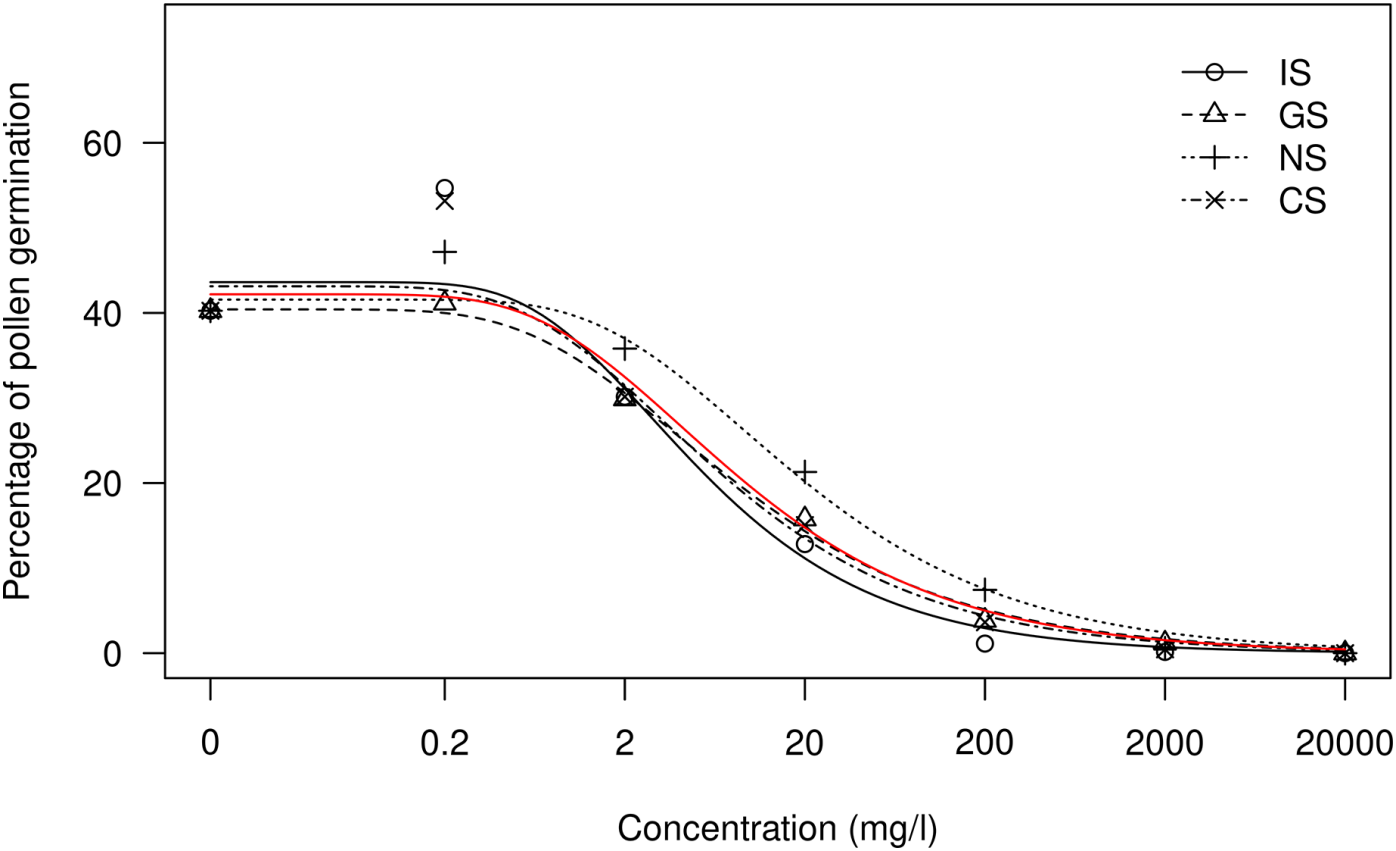
Comparison of the dose-response treatments for the Nova citrus variety. Each point represents the mean value for every treatment and concentration. The red line represents the model for all the treatments together. Treatments: BK, positive control; CS, copper sulfate; NS, ammonium sulfate; IS, inorganic sulfur; GS, water dispersible granular sulfur.

On the other hand, the dose-response curves for the treatment comparison showed a similar trend for all the treatments at each sulfur concentration (Figs. 3 and 4). Therefore, the response depended more on the total sulfur concentration than on the way in which IS is applied as GS, NS or CS, as mentioned above. The dose-response curves for Nova tangelo showed similar trends in the four sulfur treatments (Fig. 4), whereas greater differences were observed for Clemenules mandarin (Fig. 3). However, for both varieties, the NS treatment was the least effective and the IS treatment was the most effective.

Regarding the inhibition effect, over 96% inhibition was achieved for the treatments with 2,000 and 20,000 mg l^−1^ of sulfur (Table 3). Inhibition was around 50% in the treatments with 20 mg l^−1^ of sulfur (SM1). In Clemenules, differences in treatments were clearly observed when the curve model was considered for the pollen germination rate per applied dose (Fig. 3). The treatments showing the most evident effects were IS, the most toxic, and NS, the least toxic, in both tested varieties. The results obtained for Nova were similar (Fig. 4), for which the most toxic treatment was IS and the least toxic one was NS.

**Table 3:** Inhibition percentage of pollen germination on Clemenules and Nova citrus varieties in relation to the control for the four sulfur-based products and the six tested concentrations.

Variety	Treatment	0.2	2	20	200	2000	20000
Clemenules	CS	−4.01	13.87	36.44	92.69	100.00	99.79
Clemenules	NS	−16.01	16.58	25.50	69.34	98.88	98.93
Clemenules	IS	−34.73	23.92	62.12	98.19	100.00	100.00
Clemenules	GS	−7.76	20.01	40.28	73.59	98.22	99.77
Nova	CS	12.26	21.95	60.62	92.37	99.16	100.00
Nova	NS	20.58	9.82	48.92	78.04	98.65	100.00
Nova	IS	8.46	23.47	66.13	96.06	99.74	100.00
Nova	GS	31.69	21.09	54.26	87.92	96.29	100.00

**Note:**

Treatments: CS, copper sulfate; NS, ammonium sulfate; IS, inorganic sulfur; GS, water dispersible granular sulfur.

**Figure 5:**
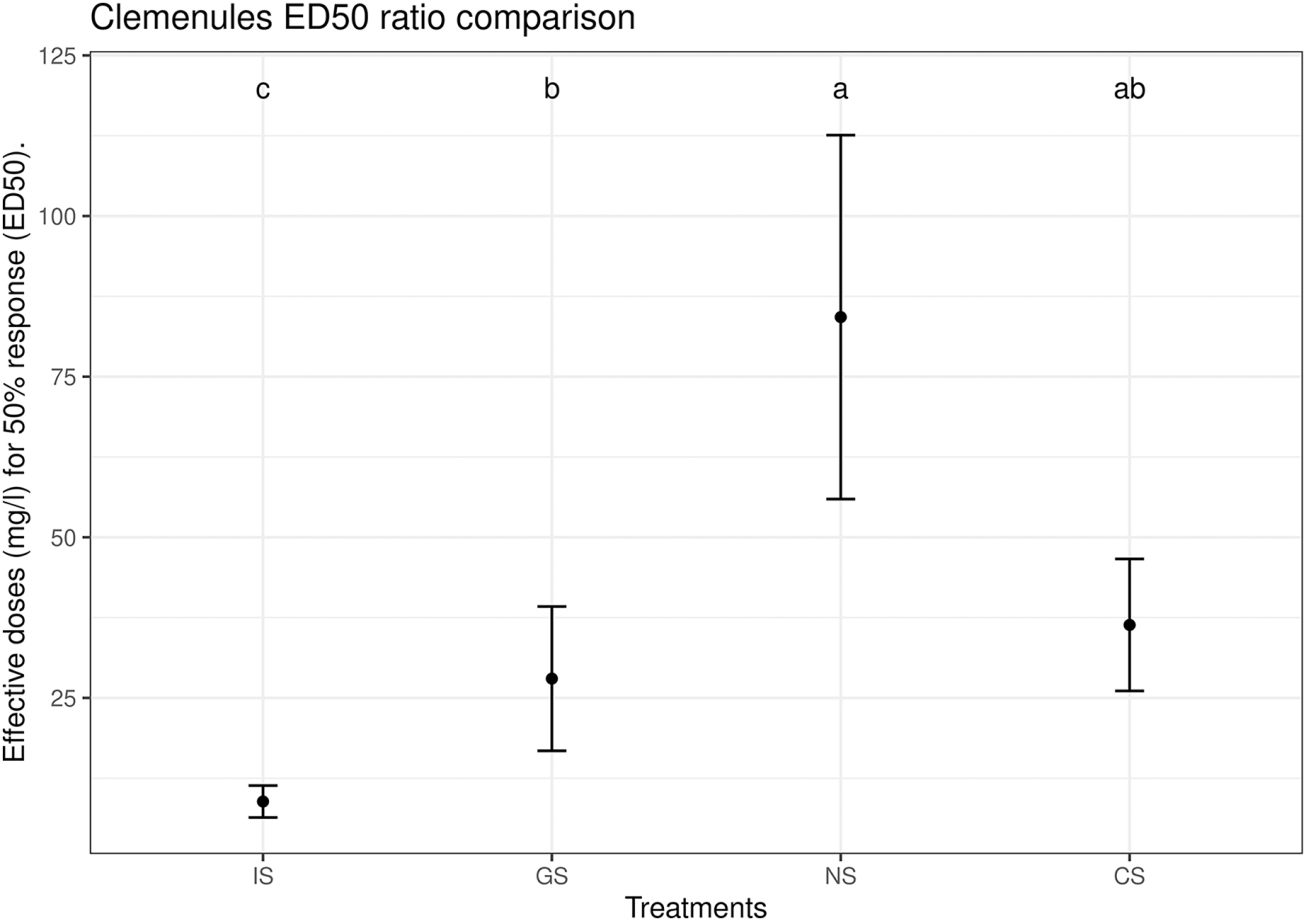
Estimated CTC50 (median effective dose) parameter for the different treatments in the Clemenules citrus variety with the Weibull-I model and three parameters. The center point is the estimation of the CTC50 value, and the error bars represent the 95% confidence interval. Treatments: BK, positive control; CS, copper sulfate; NS, ammonium sulfate; IS, inorganic sulfur; GS, water dispersible granular sulfur.

**Figure 6:**
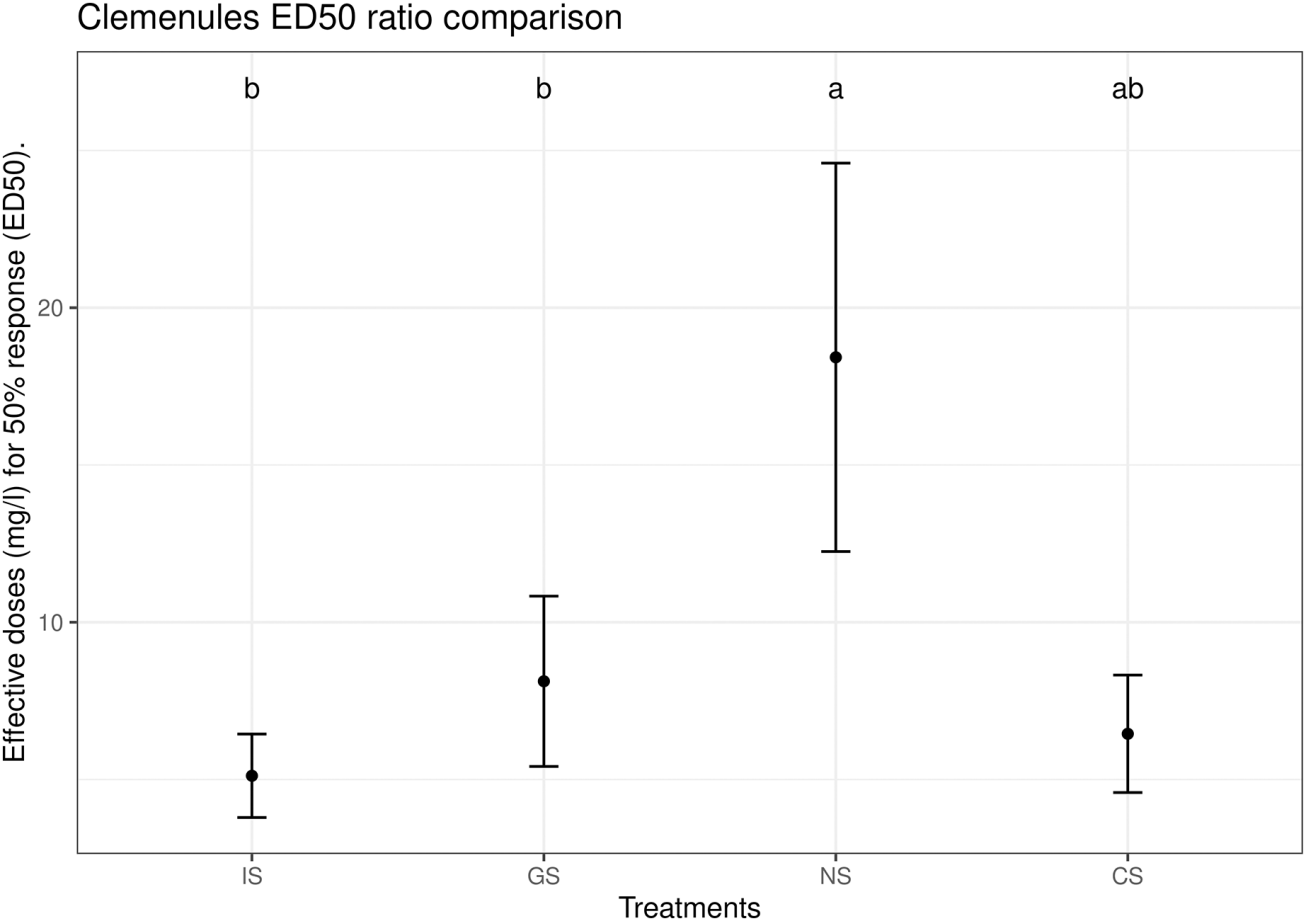
Estimated CTC50 (median effective dose) parameter for the different treatments in the Nova citrus variety with the Weibull-I model and three parameters. The center point is the estimation of the CTC50 value, and the error bars represent the 95% confidence interval. Treatments: BK, positive control; CS, copper sulfate; NS, ammonium sulfate; IS, inorganic sulfur; GS, water dispersible granular sulfur.

In more detail, the sulfur dose that had an exact inhibition effect on 50% of the population (CTC50, median effective dose) was calculated for each variety and treatment. For Clemenules, CTC50 ranged from 11.5 mg l^−1^ for IS to 85.5 mg l^−1^ for NS with significant differences between them (Fig. 5), For Nova, CTC50 ranged from 5.5 mg l^−1^ for IS to 23.7 mg l^−1^ for NS, also with significant differences between them (Fig. 6). The CTC50 in all the tested sulfur-based products was around 20 mg l^−1^. All this indicates a cumulative toxic effect of sulfur regardless of the type of sulfur-based product used, which increased as the sulfur concentration rose. In any case, wide variability was found in the NS results for both varieties. No significant differences were observed between CS and the treatments with the highest CTC50 and the lowest CTC50 for any variety. Finally, only in the case of Clemenules GS took an intermediate position between IS and NS.

## Discussion

Clear inhibition of pollen tube germination was observed in all the tested sulfur-based treatments (IS, GS, NS and CS). There were no significant differences between treatments, although the inhibition effect observed for inorganic sulfur was greater than that for the other products. The effect of sulfur on the inhibition of pollen germination of Clemenules mandarin and Nova tangelo depends on the dose, and not in the way in which this element has been formulated. To the best of our knowledge, no previous studies report the effect of inorganic sulfur on pollen germination. However, several studies indicate the effect of atmospheric sulfur dioxide on pollen. Pollen germination inhibition by sulfur dioxide is demonstrated in a study performed on *Pinus syvestris* L. in Poland (Mejnartowicz & Lewandowski, 1985). Other forest trees like *Populus* also show loss of fertility and less pollen tube growth after sulfur dioxide application (Venne, Scholz & Vornweg, 1989). Several of these papers also talk about the importance of the dose to correctly observe the effect. Specifically, Ouyang et al. (2016) studied the effect of sulfur dioxide at different doses on oak pollen, pointing out that low doses can cause deformations in the pollen grains while higher doses would cause the total degradation of the pollen. No references were found about sulfur dioxide affecting the pollen of *Citrus* species, although the effect of this substance on the inhibition of pollen of other cultivated trees like apple has been reported (Munzuroglu, Obek & Geckil, 2003).

On the studied inorganic sulfates, we have no prior knowledge about the effect of NS, but there are several studies on CS. Mesejo et al. (2006) stated that the CS concentration of 25 mg l^−1^ is capable of partially affecting the pollen of ‘Fortune’ mandarins by inhibiting pollen germination during *in vitro* tests. The above-cited study indicates that *in vivo* CS application to ‘Clemenules’ and ‘Afourer’ mandarin flowers avoids fertilization and seed formation. These results agree with the result observed in the present study because a CS concentration over 200 mg l^−1^ inhibited the germination of more than 90% of pollen grains compared to the control. Gambetta et al. (2013) performed a study in Uruguay on ‘Afourer’ mandarins and verified that applying CS at a rate of 125 mg l^−1^ did not appreciably decrease the number of seeds per fruit, which revealed that total pollen germination inhibition did not occur. In these two previous studies, the authors attributed possible pollen germination inhibition to the action of copper rather than to sulfur. However, our study suggested that partial pollen germination inhibition was caused mainly by sulfur and not by copper.

Many references appear in which several compounds with sulfur, employed as a common chemical element, had a clear effect on pollen germination, while very little evidence for copper is available. Only a slight effect on pollen germination and pollen tube growth has been observed in quince (*Cydonia oblonga* L.) and plum (*Prunus domestica* L.) after applying copper chloride, while more powerful pollen germination inhibition has been found for other heavy metals, such as cadmium or mercury (Topdemir & Gür, 2005). In a study conducted in an area with mining activity, some pollen alterations in wild plant species have been noted, but without specifying if this was due to the accumulation of iron, copper or other metals (Yousefi et al., 2011). The results herein obtained suggest that sulfur is the active element because all the sulfur-based treatments significantly reduced the pollen germination of the Clemenules and Nova varieties.

There are no references about the effect of IS applications on anthers or pollen grains of *Citrus* species, although some similar studies for other crops can be found. An experiment was carried out on apple (*Malus domestica* L.) by applying liquid lime sulfur at a rate of 20,000 mg l^−1^ combined with fish oil. The authors observed total pollen tube growth inhibition in the style when lime sulfur was applied 4 or 24 h after pollination (Yoder et al., 2009). Evidence for a generic effect of sulfur acting on the pollen germination process and pollen tube development inside the style, and on subsequent ovule fertilization, is becoming clearer. As noted above, several sulfur-based products prevent normal pollen activity in cultivated, wild or forest plants. Garmendia et al. (2022a) tested the effect of sulfur in an *in vivo* test on ‘Nadorcott’ mandarin flowers by applying 5,000 mg of pure solid elemental sulfur (the equivalent to the IS herein used) to each stigma. These authors verified total pollen germination inhibition after applying sulfur, regardless of whether flower pollination occurred 24 h earlier or 24 h later. Their study suggests that the effect of sulfur on that citrus variety is generic and permanent. Our present results also agree with these results. In fact, it can be inferred that IS probably affects the pollen development of any *Citrus* species or varieties. The practical application of these results can be very extensive. One of those applications can be to obtain seedless citrus fruits in traditional seedy varieties. In fact, Garmendia et al. (2022b) indicated recently that field applications of sulfur in ‘Nadorcott’ trees produced a significant decrease in the number of seeds per fruit, with a reduction of up to 87% of seed compared to the control. In any case, further research must be conducted to better explore this possibility and find out the appropriate moments of application, the dose used, *etc*., especially considering that the dose is an important factor for the effect of pollen inhibition, as we have observed in this work.

## Conclusions

The four sulfur-based treatments (IS, GS, NS and CS) inhibited the pollen grain germination of two varieties (Clemenules and Nova). Inhibition did not occur at the lowest concentration (0.2 mg l^−1^) but was total for the highest sulfur concentrations (2,000 and 20,000 mg l^−1^). Inhibition was around 50% in the treatments with 20 mg l^−1^ of sulfur. The observed effect of sulfur in both mandarin Clemenules and tangelo Nova depends on the dose and not on the formulation. Although no significant differences were observed between the four products, inorganic sulfur showed the highest percentage of inhibition. This is the first evidence of the effect of inorganic sulfur on the pollen inhibition of citrus varieties. This effect could have important applications in agronomy, such as obtaining seedless fruits in any variety of citrus. In any case, further research is required to demonstrate the inhibition of pollen *in vivo*, and to find out what role the stigma of the flower may have in the whole process.

## Supplemental Information

10.7717/peerj.14775/supp-1 Supplemental Information 1 Inhibition percentage of pollen germination for the six tested concentrations with Nova and Clemenules citrus varieties. Treatments: BK, positive control; CS, copper sulfate; NS, ammonium sulfate; IS, inorganic sulfur; GS, water dispersible granular sulfur.

10.7717/peerj.14775/supp-2 Supplemental Information 2 Raw data of pollen germination for Clemenules and Nova varieties. Treatments: PAP (IS), inorganic sulfur; PAWG (GS), water dispersable granbular sulfur; PANH (NS), ammonium sulfate; PACU (CS), copper sulfate; BK, standard germination medium (positive control)

## Additional Information and Declarations

### Data Availability

The following information was supplied regarding data availability: The raw data of all plates treated with the four sulfur-based products cited in the article are available in the Supplemental File.
