# Supplementary figures and images for: Effect of sulfur on pollen germination of Clemenules mandarin and Nova tangelo
Source: PeerJ. 2023 Feb 7;11:e14775. doi: 10.7717/peerj.14775 (PMC9912945; doi:10.7717/peerj.14775)

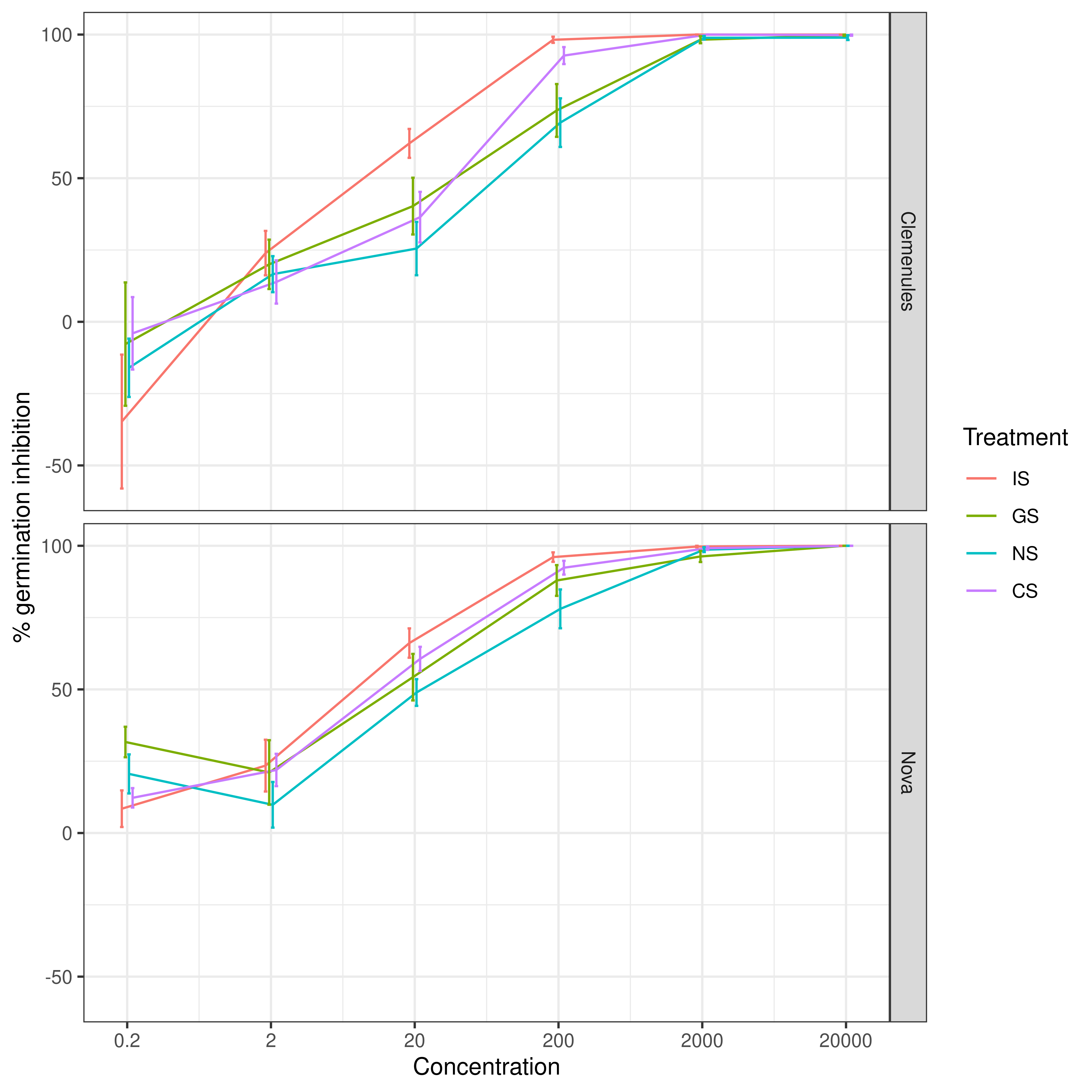

Supplement: Supplemental Information 1 — Treatments: BK, positive control; CS, copper sulfate; NS, ammonium sulfate; IS, inorganic sulfur; GS, water dispersible granular sulfur. [file peerj-11-14775-s001.png]
